# Dynamics of Perceived Barriers to Change Physical Activity and Eating Habits in Adults Before and During a Lifestyle Intervention in a Primary Care Setting: A Mixed Methods Approach

**DOI:** 10.1177/21501319241273321

**Published:** 2024-08-15

**Authors:** Fawzi Kadi, Andreas Nilsson

**Affiliations:** 1 School of Health Sciences, Örebro University, Örebro, Sweden

**Keywords:** healthy diet, behavioral change, physical exercise, prevention, health promotion

## Abstract

**Background:**

There is scarcity of data exploring the dynamics of barriers to changing physical activity (PA) and eating habits during a lifestyle intervention in a primary care setting. The aim of the present study was to investigate barriers to lifestyle change before and during a primary care lifestyle intervention in adults with different sociodemographic backgrounds.

**Methods:**

Barriers to healthy eating and PA were assessed in 114 adults (age = 55 ± 9 years) using a questionnaire before inclusion in the intervention. During the lifestyle intervention, factors perceived as obstacles to reach goals for PA and healthy eating habits were collected using focus group interviews in a sub-sample of 25 adults and analyzed using thematic analysis.

**Results:**

At least 1 barrier to changing eating and PA habits was reported in 65% and 67% of the population, respectively, without differences due to sex, place of birth, and educational level. Before entering the lifestyle intervention, intrapersonal factors including lack of willpower and lack of enjoyment were the dominant barriers to PA and healthy eating, irrespective of place of birth, sex, and education level. In contrast, environmental factors such as lack of support from family and work-related constraints emerged as important barriers to overcome during the action phase of the lifestyle intervention.

**Conclusions:**

The present study highlights the dynamics of barriers to changing PA and eating habits in a primary care lifestyle intervention, emphasizing the need for barrier assessment during the different phases of an intervention to tailor support activities for successful lifestyle behavioral change.

## Background

Poor eating habits and low physical activity (PA) level are key modifiable risk factors with detrimental effects on general health, including physical and psychological health outcomes as well as quality of life.^1,2^ Unfortunately, physical inactivity remains one of the leading risk factors for development of chronic diseases, with about a quarter of the world’s population being insufficiently active.^3^ Similarly, up to a quarter of all deaths among adults are associated with poor dietary habits, with no major improvements in intakes of fruit and vegetables and whole-grains during the past decade.^4^ Thus, there is an urgent need to further develop strategies aiming to improve adherence rates to healthy lifestyles. Such strategies should integrate the role of socioeconomic status as an important determinant of lifestyle behaviors^5^ as well as risk of chronic disease.^6^ Indeed, social attributes related to the surrounding neighborhood (eg, safety issues, deprivation, and lack of community cohesion) alongside individual factors such as education level are associated with increased disease risk.^6-8^

The primary care has the potential to be an important arena for disease prevention through promotion of physical activity and healthy eating habits targeting adults at high disease risk across different socioeconomic backgrounds.^9^ Indeed, there is convincing evidence indicating that well organized primary care systems are associated with a better population health and a more equitable distribution of health across citizens.^10,11^ However, the delivery of lifestyle preventive measures in primary care remains scarce^12^ and healthcare practice is mainly directed toward disease management.^13^ Therefore, the identification of barriers to lifestyle change is instrumental for supporting the development of prevention models in primary care settings.^14,15^ Such barriers are generally related to intrapersonal and environmental factors, where individuals may experience different types of barriers to healthy lifestyle behaviors due variations in sociodemographic backgrounds.^16,17^ In this respect, lifestyle intervention programs based on behavioral models such as the health action process approach (HAPA)^18^ are generally designed to empower individuals to overcome perceived intrapersonal and environmental obstacles to PA and healthy eating. Previous reports have identified several important factors interfering with the adoption of healthy eating habits, including lack of knowledge about healthy food choices, lack of cooking skills, or lack of willpower to change dietary habits.^19,20^ Likewise, several factors considered as obstacles to becoming more physically active have been identified, including lack of exercise opportunities and time constraints.^21-23^ Importantly, perceived barriers to lifestyle change are frequently assessed at 1 time point before entering an action phase of a lifestyle intervention. However, barriers to lifestyle change may vary during the process of forming behavior intention to translation into engagement in the planned health behavior. Currently, we are unaware of any published data exploring the dynamics of perceived barriers to changing both PA and eating during a lifestyle intervention in a primary care setting. Therefore, the aim of the present study was to investigate perceived barriers to changing PA and eating habits before and during a primary care lifestyle intervention in adults with different sociodemographic backgrounds.

## Methods

### Participants

A total of 114 adults were screened for potential inclusion in a randomized controlled trial aiming to promote physical activity and healthy eating habits. Recruitment of participants was performed using advertisement at the primary care unit and the surrounding area, as well as by mailing to recipients living in surrounding area. The intervention was conducted in a Swedish primary care covering adults with different sociodemographic characteristics. A written informed consent was obtained from participants and all investigations were performed in accordance with the Declaration of Helsinki ethical principles. The Swedish Ethical Review Authority approved the study (Dnr 2022-02435-02) and the trial protocol is registered at ClinicalTrials.gov (NCT05689762).

### Perceived Barriers to Changing PA and Eating Habits Before Entering the Lifestyle Intervention

Barriers to healthy eating and PA were assessed in the screening phase prior to inclusion in the behavioral change trial. All participants (n = 114) reported on barriers to PA and healthy eating using questionnaires based on previous work, where participants were asked to select up to 3 of the most important perceived barriers to lifestyle change.^20,22,24^ The questionnaires consisted of a list of potential barriers, together with the possibility to add barriers not listed.

### Description of the Primary Care Lifestyle Intervention

During screening of participants, data on biological sex, place of birth, marital status, educational level, retirement status were assessed by self-report. Participants with an age between 40 and 70 years old and with elevated disease risk were included in the lifestyle intervention. Elevated disease risk was defined according to the following: poor lifestyle habits (not meeting guidelines for PA and/or dietary habits), together with waist circumference above sex-specific cut-points and/or BMI of at least 25 and/or blood glucose and blood lipid levels above established cut points for elevated metabolic risk. Participants were randomized to intervention and control arms. The intervention arm received health counseling aiming to promote physical activity and healthy eating habits delivered by 2 health professionals. The focus of the behavioral change counseling was to support motivation to change (motivational phase) by addressing risk perception, outcome expectancy and action self-efficacy, and to support the translation of intention into action (volitional phase) by strengthening action planning and maintenance self-efficacy according to the HAPA model.^18^

### Perceived Barriers to Changing PA and Eating Habits During the Lifestyle Intervention

Three weeks after initiation of the action phase of the lifestyle intervention, a subgroup of 25 participants were prompted to share their experiences about factors perceived as obstacles for becoming physically active and making healthier food choices. These experiences were explored using focus group interviews performed in groups of 6 to 7 participants. The focus group interviews were selected to better capture experiences after receiving the behavioral change counseling to provide an in-depth understanding of perceived barriers during a behavioral change process. Participants were asked to reflect about perceived barriers based on the following main questions “which factors have you experienced as barriers for becoming more physically active?” and “which factors have you experienced as barriers for making healthier food choices?”. After multiple readings of interview transcripts by 2 investigators, sections of text were extracted verbatim. Thematic analysis was used to identify perceived barriers to changing PA and eating habits by first labeling meaningful quotes and thereafter generating sub-themes.^25^ The various sub-themes were further grouped into themes representing the overall type of barrier.

### Statistical Analysis

Quantitative data on barriers to changing diet and PA habits are reported as percentages. Differences in frequency of reporting at least 1 barrier to diet and PA, respectively, between groups based on sex, marital status, educational level and place of birth were analyzed using chi-square (χ^2^) testing. Alpha was set to *P* = .05.

## Results

A total of 114 participants (age = 55 ± 9 years) were screened for inclusion in the lifestyle intervention. Characteristics of the participants are presented in Table 1. All 114 participants reported on perceived barriers to changing PA and eating habits prior to inclusion in the lifestyle intervention. Of those randomized to the intervention group, a subgroup of 25 participants reported on perceived barriers after entering the action phase of the lifestyle intervention using focus group interviews. The demographic characteristics of this subgroup were as follows: 52 % were women, 40% were born outside Sweden, 32% were living alone, 48% had an education level below college-University level and 44% were retired.

**Table 1.** Subject Characteristics.

	All (n = 114)
Sex	
Male (n)	42
Female (n)	72
Place of birth	
Scandinavian area (n)	72
Outside Scandinavian area (n)	42
Educational level	
Primary/secondary school (n)	56
College/university (n)	58
Marital status	
Married/living together (n)	83
Living alone (n)	31
Retirement status	
Retired (n)	25
Not retired (n)	89

### Perceived Barriers to Changing PA and Eating Habits Before Entering the Lifestyle Intervention

**Figure 1.**
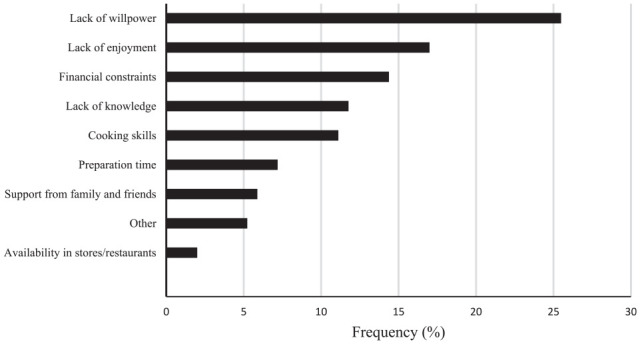
Barriers to changing eating habits.

Approximately two-thirds of the population reported at least 1 barrier to healthy eating (65%) and PA (67%). There were no significant differences in the likelihood of reporting barriers to either PA or diet across the sociodemographic variables sex, place of birth, and educational level. Compared to those living with a partner, living without a partner was related to a significantly (*P* < .05) higher likelihood of reporting barriers to diet but not to PA. Barriers to healthy eating are presented in Figure 1. Based on the whole sample, the 3 most frequently reported barriers were the intrapersonal factors lack of willpower and lack of enjoyment, together with the environmental factor financial constraints (Figure 1). Notably, besides these common barriers, cooking skills and lack of knowledge about how to eat healthier also emerged as prominent intrapersonal barriers to change eating habits across sociodemographic variables.

**Figure 2.**
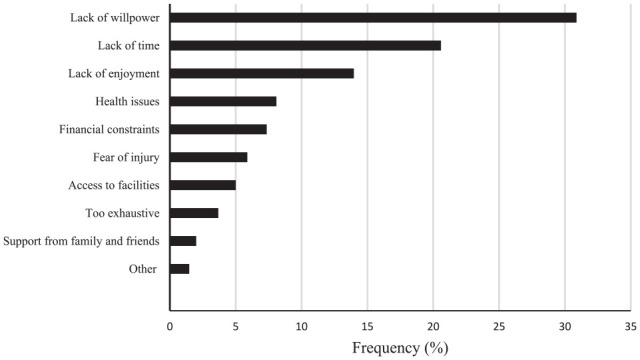
Barriers to changing physical activity habits.

Barriers to changing PA habits in the whole population are presented in Figure 2. The 3 most frequently reported barriers were the intrapersonal factors lack of willpower, lack of time, and lack of enjoyment. In addition to these barriers, health-related issues and financial constraints were among the 3 most frequently reported barriers in those with lower education level and retired participants, respectively.

Notably, before entering the primary care lifestyle intervention, family support, or availability of healthy foods in stores and restaurants and access to PA facilities were not frequently reported as barriers to changing PA and eating habits.

### Perceived Barriers to Changing PA and Eating Habits During the Lifestyle Intervention

In contrast to information collected before entering the lifestyle intervention, thematic analysis based on transcripts from focus group interviews showed a shift in perceived barriers with an emphasis on environmental rather than intrapersonal factors as the dominant barriers.

Environmental factors included the following sub-themes:

*1. Lack of family support.* Participants experienced several issues related to the family situation as a barrier to adopting healthy lifestyle habits. Examples of statements hampering healthy eating were: “*I tried to eat more vegetables. . . but I don’t want to remove high-fat dairy products as my son needs it*,” “*my partner and children may not accept that I cook more root-vegetables*” or “*when I cook, I have to think about what the whole family likes to eat.*” Examples of statements hampering increasing amount of physical activity were: “*it is difficult to change my physical activity habits when having children*,” or “*children think that walking for exercise is boring.*”
*2. Financial constraints.* The costs of healthy foods were perceived as a barrier to healthy eating. Examples of statements included “*I would like to eat more fish, but my economy does not allow it*” and “*It is expensive. . .especially when food goes to waste since my children don’t like healthy foods.*”
*3. Work-related situation.* The job situation emerged as a barrier to becoming more physically active. Examples of statements are: “*I sit all day at work, so I have little opportunity to be more active*” or “*I have a physically demanding job and I don’t have the energy to be physically active at home.*” Employments involving traveling were also perceived as a barrier to make healthy food choices. An example of statement is: “*on a work-related travel, it is difficult to eat healthy, one likes to drink a beer and eat a sandwich for dinner.*”
*4. Surrounding neighborhood.* Feelings of being unsafe in the surrounding neighborhood were raised as a barrier to outdoor exercise activities. An example of statements is: “*I hear a lot of bad things happening in the neighborhood, so I don’t dare to exercise outside alone.*”
*5. Weather conditions*. Weather conditions were perceived as a factor limiting opportunities for outdoor exercise activities. An example of statement is: “*this time of the year, the weather conditions does not allow me to exercise outdoors the way I want.*”

Intrapersonal factors included the following sub-themes:

*1. Health-related issues*. Physical function limitation was raised as a barrier to PA. An example of statement is: “*I would like to go swimming, but I have pain in my arms.*”
*2. Lack of time.* The feeling of lacking time for changing lifestyle habits was expressed. Examples of statements were: “*I would like to do more physical activities, but time is sparse*” or “*It takes more time to cook healthy food.*”

## Discussion

The present study aimed to investigate perceived barriers to changing PA and eating habits determined before and during transition into the action phase of a lifestyle intervention in a primary care setting. While barriers related to intrapersonal factors, such as lack of willpower and lack of enjoyment were dominant obstacles to lifestyle change before entering the action phase, environmental factors such as lack of support from family and work-related constraints emerged during the transition to the action phase. These findings suggest the need for barrier assessment during the different phases of a lifestyle intervention to successfully support behavioral change.

Prior to entering the action phase of the lifestyle intervention, lack of willpower and lack of enjoyment were the most frequently perceived barriers to PA and healthy eating, together with financial constraints for healthy eating and lack of time for PA, respectively. This is in line with previous observations indicating these barriers as key to overcome for adoption of a physically active lifestyle and healthy eating across adult populations, with small variations due to sociodemographic factors.^26-30^ This finding also denotes the importance of including behavioral change techniques (BCTs) aiming to empower individuals to overcome intrapersonal barriers before entering the action phase. In this respect, increasing health literacy and self-efficacy, individualized goal setting and planning of the behavior change are BCTs with potential to improve uptake of healthy behaviors.^31^ Notably, lack of cooking skills was perceived as a barrier in those living without a partner. Therefore, educational activities supporting increased food literacy and information about low-cost healthy foods together with practical cooking demonstrations may represent specific activities that need to be addressed in a lifestyle intervention. Alongside the major barriers to PA, health-related issues and financial constraints emerged as additional factors that may limit adherence to PA goals, which suggests the need to individualize counseling about opportunities to engage in physical activities of different types and settings to increase adherence to PA guidelines.

Data generated after entering the action phase of the intervention revealed an accentuated importance of environmental factors perceived as barriers, including family support, work-related environment for PA and eating behaviors, as well as weather conditions for PA only. Notably, making healthier food choices may not be accepted by other family members, creating an obstacle to healthy eating. To overcome this family-related barrier, which has previously been reported in several studies,^32^ strengthening confidence and creativity in meal preparation and involving other family members in meal planning and meal preparation may facilitate changes in eating habits.^33^ Similarly, occupational-related sedentary behavior requires action plans incorporating several strategies, including active commute to work, breaking prolonged sedentary behavior at the workplace and increasing leisure-time moderate-to-vigorous PA. Establishing a set of opportunities for being physically active would also favor adherence to healthy lifestyles regardless of weather conditions. Noteworthy, feelings of being unsafe related to the surrounding neighborhood was raised as an additional environmental factor acting as barrier to become more physically active. Therefore, group-based physical activities together with involvement of local community organizations may facilitate adoption of healthy lifestyle habits when targeting individuals living in socioeconomically disadvantaged areas. While environmental factors are commonly acknowledged as key barriers to overcome,^14,21^ less emphasis has been put on the dynamics of perceived barriers to lifestyle change in relation to different phases of a behavioral change process. In this primary care lifestyle intervention, a shift in factors perceived as major obstacles for behavior change emerged, with intrapersonal factors perceived as prominent barriers before entering the lifestyle intervention, while environmental factors were highlighted as important barriers to overcome after transition to the action phase. The shift in perceived barriers to lifestyle change over time likely reflects changes in mindsets that occur during the transition from formation of behavior intention to engagement in the actual health behavior.^18^ According to the HAPA theoretical framework, an individual’s mindset when preparing a behavior change differs from that when engaging in the new health-enhancing behavior.^18^ Thus, the shift in barriers to lifestyle change indicates that translation of intentions into new behaviors requires a new set of supporting measures for successful adoption and maintenance of healthy lifestyles. Hence, it is suggested that intrapersonal barriers are emphasized during the intentional phase, whereas environmental barriers are addressed during the subsequent action phase. The shift in perceived barriers to lifestyle change indicated in our study is supported by another report revealing changes in the number and type of barriers to PA during a lifestyle intervention.^34^ This strengthens the need to monitor barriers at different phases of a lifestyle intervention to better tailor behavioral techniques supporting adoption and long-term maintenance of healthy behaviors.

The present study has strengths and limitations. A strength of the present study is that barriers to lifestyle change were assessed in a sample of adults with different sociodemographic characteristics. Another strength of the study is that barriers were assessed in different phases of a behavioral change process, which can be used to inform design of future primary care lifestyle intervention programs. However, the study is not without limitations. First, given that all the information on perceived barriers to lifestyle change were self-reported, bias due to social desirability cannot be ruled out. Second, caution should be taken before generalizing the study findings to broader populations and wider intervention contexts. In particular, content and delivery of the intervention may likely impact on perceived barriers to healthy lifestyle change. Therefore, further research on the dynamics of barriers to lifestyle change is needed to guide delivery of future intervention efforts.

## Conclusion

In conclusion, the present study highlighted the dynamics of perceived barriers to changing PA and eating habits in a primary care lifestyle intervention, which emphasizes the need for barrier assessment during the different phases of a lifestyle intervention to tailor support activities for successful lifestyle behavioral change.
